# Supplementary material for: A taxonomic reassessment of Piramys auffenbergi, a neglected turtle from the late Miocene of Piram Island, Gujarat, India
Source: PeerJ. 2018 Nov 15;6:e5938. doi: 10.7717/peerj.5938 (PMC6240434; doi:10.7717/peerj.5938)
Supplement: Supplemental Information 2 [file peerj-06-5938-s002.pdf]

Supplementary File S2 for

A taxonomic reassessment of *Piramys auffenbergi*, a neglected turtle from the late Miocene of Piram Island, Gujarat, India

Gabriel S. Ferreira, Saswati Bandyopadhyay, Walter G. Joyce
correspondece to: Walter G. Joyce, walter.joyce@unifr.ch, walter.g.joyce@gmail.com

This PDF file includes additional information of the phylogenetic analyses

1. List of changes to Ferreira et al. (2018)'s matrix
2. Strict consensus tree with node numbers
3. Bootstrap, Jacknife & Bremer support information
4. List of common synapomorphies for Stereogenyina

1. Changes to Ferreira et al. (2018)'s matrix

Char. 5: PF, preorbital skull broad

Scoring: *Lemurchelys diasphax* – 1 > 0
Stereogenys cromeri – 1 > 0
Shweboemys pilgrimi – 1 > 0
Brontochelys gaffneyi – 1 > 0

Char. 9: FR, interorbital groove

Scoring: *Mogharemys blackenhorni* – 0 > ?
Brontochelys gaffneyi – 0 > ?

Char. 12: FR, prefrontal/frontal

Scoring: *Bairdemys sanchezi* – 1 > 0

Char. 14: Anteroventral emargination

Scoring: *Cordichelys antiqua* – 1 > 0

Char. 15: QJ, quadratojugal

Scoring: *Stereogenys cromeri* – ? > 1

Char. 19: PA, dorsal portion in relation to adductor fossa

Scoring: *Stereogenys cromeri* – 0 > ?

Char. 23: PA, PA-JU contact

Scoring: *Shweboemys pilgrimi* – 0 > ?

Char. 25: PA, parietal contacts pterygoid at base of processus trochlearis pterygoidei

Scoring: *Mogharemys blackenhorni* – 0 > 1
Brontochelys gaffneyi – 0 > 1
Lemurchelys diasphax – 0 > 1
Stereogenys cromeri – 0 > 1

Cordichelys antiqua – 0 > 1
Latentemys plowdeni – 0 > 1
Bairdemys thalassica – 0 > 1
Bairdemys sanchezi – 0 > 1
Bairdemys hartsteini – 0 > ?
Bairdemys venezuelensis – 0 > ?
Shweboemys pilgrimi – 0 > ?

Char. 26: PA, PA-PT contact in septum orbitotemporale
Scoring: *Caninemys tridentata* – 2 > 0

Char. 27: PA, interparietal scale
Scoring: *Bairdemys sanchezi* – 1 > ?

Char. 29: PA, interparietal scale meeting medially
Scoring: *Bairdemys winklerae* – 1 > ?

Char. 41: PM, pinched snout
Scoring: *Cordichelys antiqua* – 0 > 1
Bairdemys hartsteini – 0 > 1

Char. 43: PM, protrudes cranially in lateral view beyond apertura narium interna dorsal ridge
Statement: this character refers to the premaxilla protruding beyond the dorsal ridge (in lateral view) of the apertura narium externa, not interna as originally stated.
Scoring: *Shweboemys plilgrimi* – ? > 1

Char. 57: MX, triturating surface shape
Scoring: *Brontochelys gaffneyi* – 1 > 2
Shweboemys pilgrimi – 1 > 2
Stereogenys cromeri – 1 > 2
Cordichelys antiqua – 1 > 2

Latentemys plowdeni – 1 > 2
Bairdemys venezuelensis – 1 > 2
Bairdemys hartsteini – 1 > 2
Bairdemys sanchezi – 1 > 2
Bairdemys winklerea – 1 > 2

Char. 114: EX-QU contact
Scoring: *Cordichelys antiqua* – 1 > 0
Latentemys plowdeni – 1 > 0
Bairdemys thalassica – 1 > 0
Bairdemys venezuelensis – 1 > 0
Bairdemys hartsteini – 1 > 0
Bairdemys sanchezi – 1 > 0
Bairdemys winklerae – 1 > 0

New character: *246. PM, median notch in upper jaw:* 0, notch absent, mx and pm ventral ridge runs anteroventrally, forming an anterior hook (*Peltocephalus dumerilianus*); 1, notch absent or very shallow, mx and pm ventral ridge runs anteriorly to the midline (*Euraxemys essweini*); 2, notch present, mx and pm ventral ridge runs anterodorsally to the midline (*Podocnemis unifilis*)

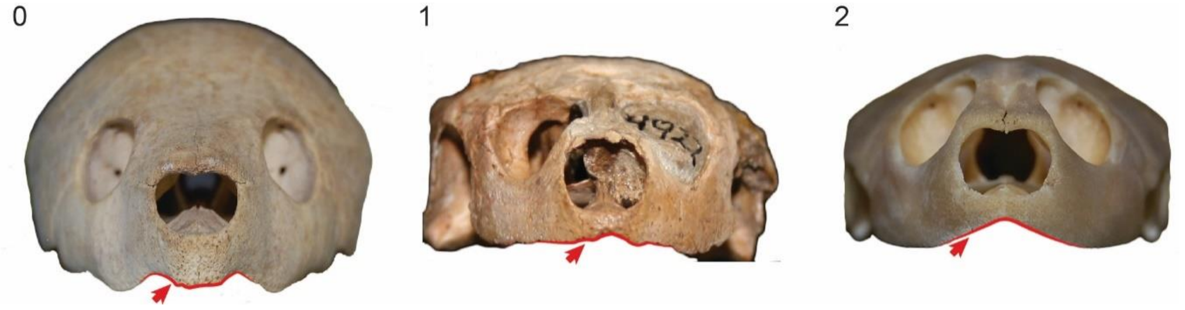

2. Strict consensus tree with node numbers

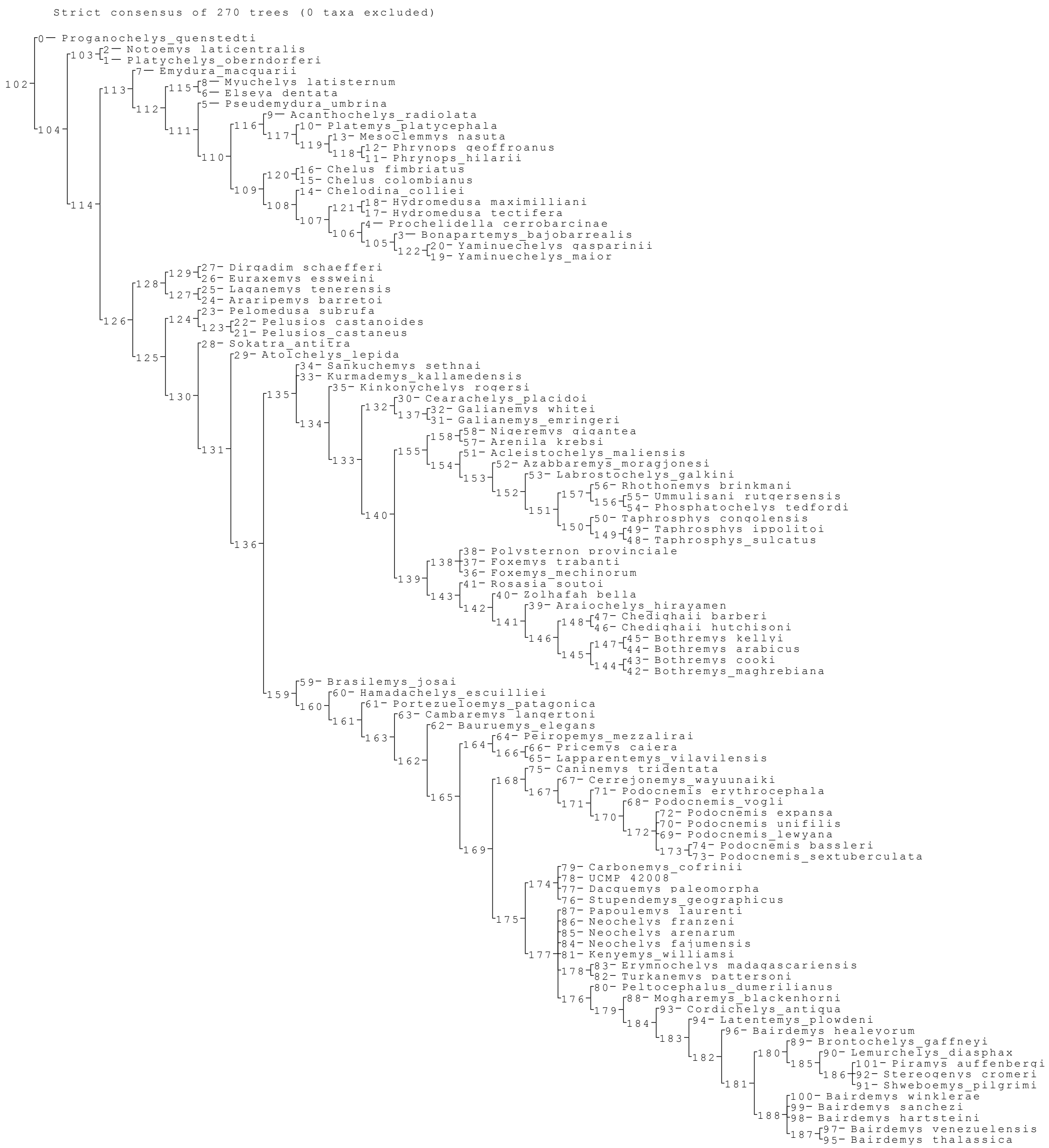

Figure S2-1. Strict consensus tree from 270 most parsimonious tress with 1134 steps

3. Bootstrap, Jacknife & Bremer support information

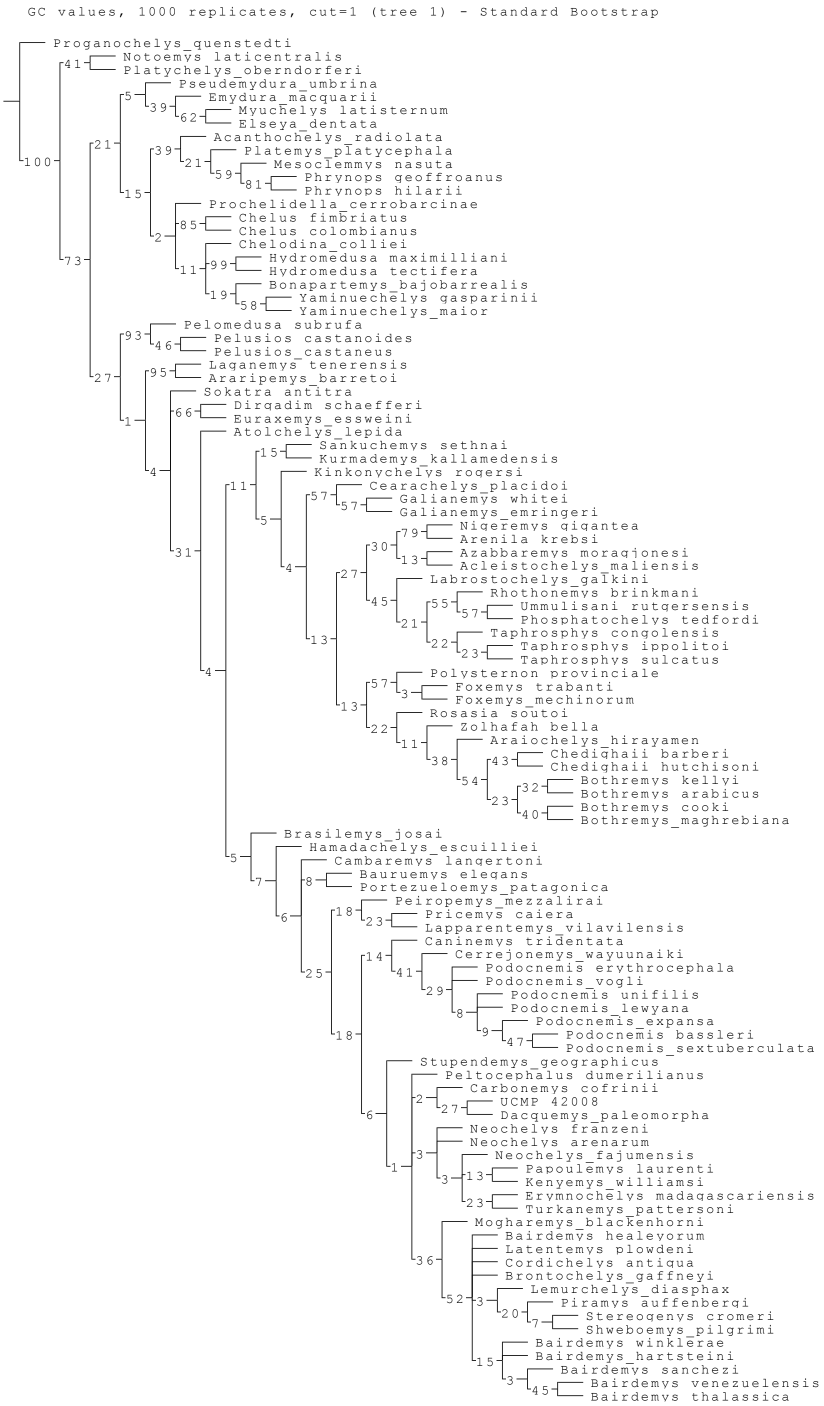

Figure S2-2. GC Bootstrap value of 1000 replicates (cut = 1)

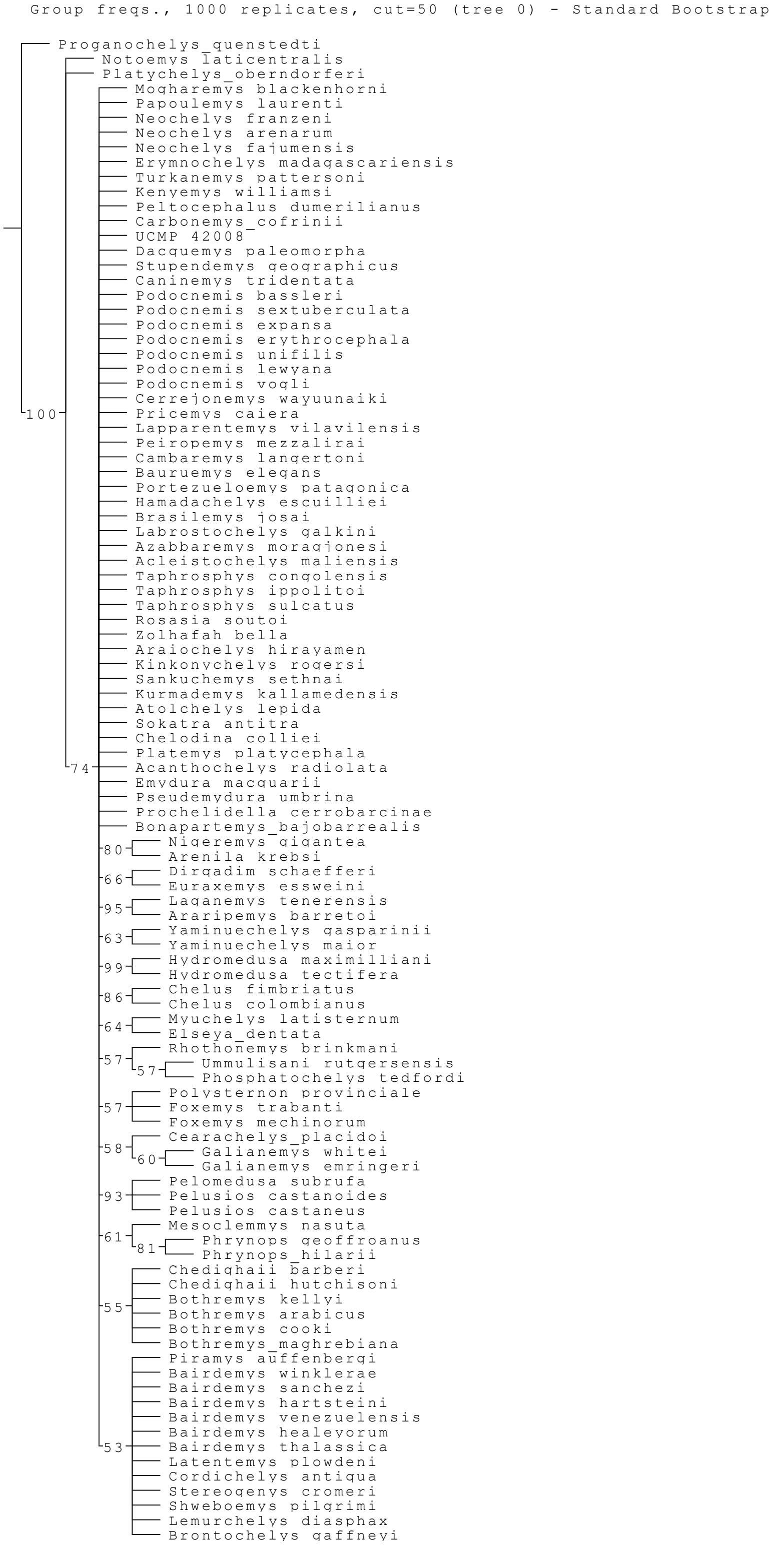

Figure S2-3. Group frequencies Bootstrap values of 1000 replicates (cut = 50)

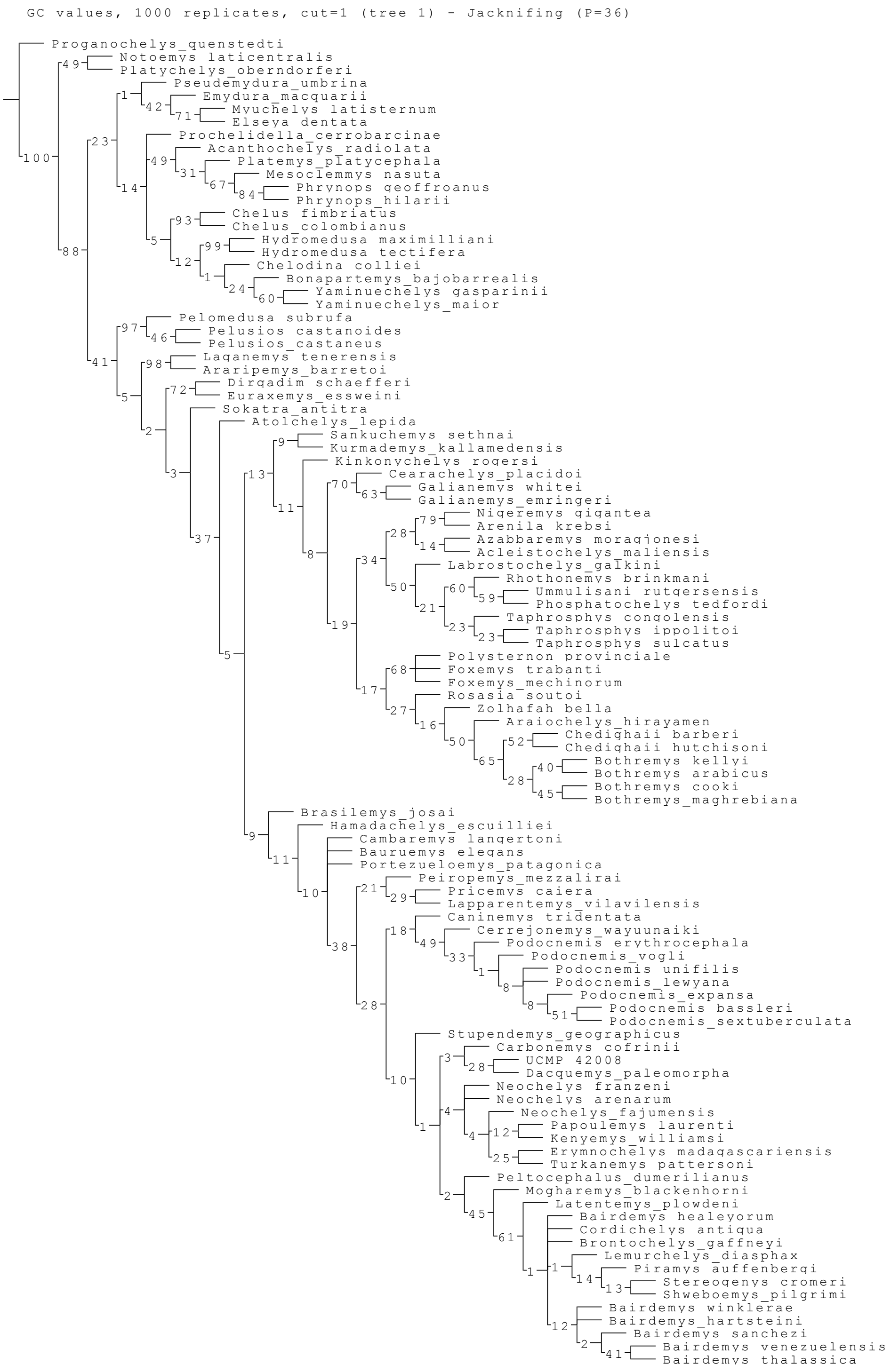

Figure S2-4. GC Jacknife values of 1000 replicates (cut = 1)

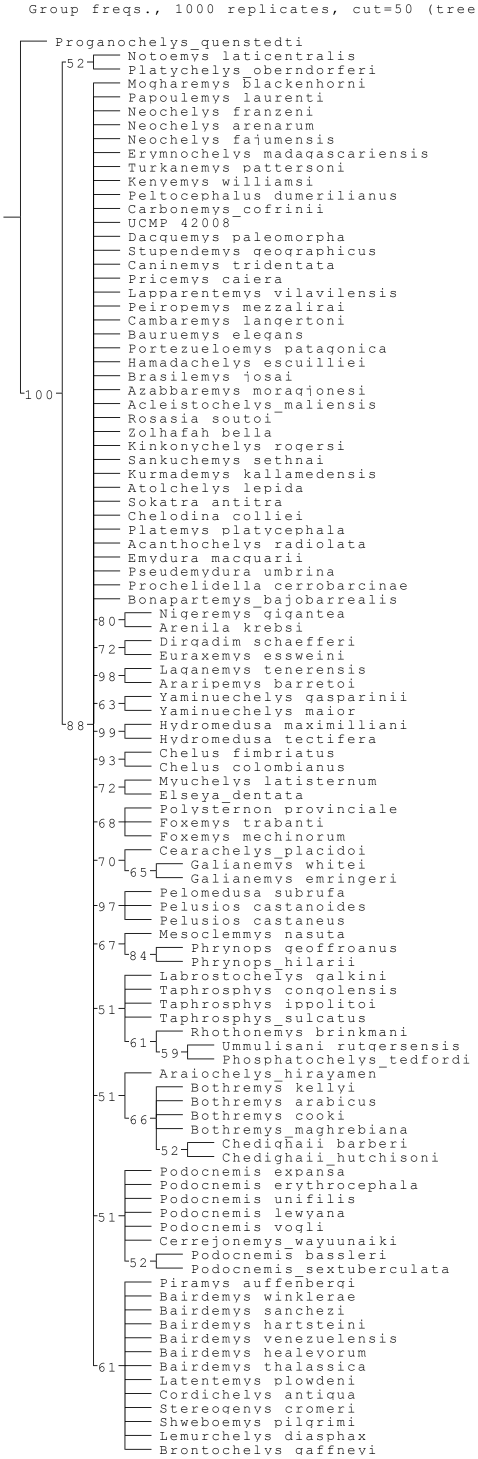

Figure S2-5. Group frequencies Jacknife values for 1000 replicates (cut = 50)

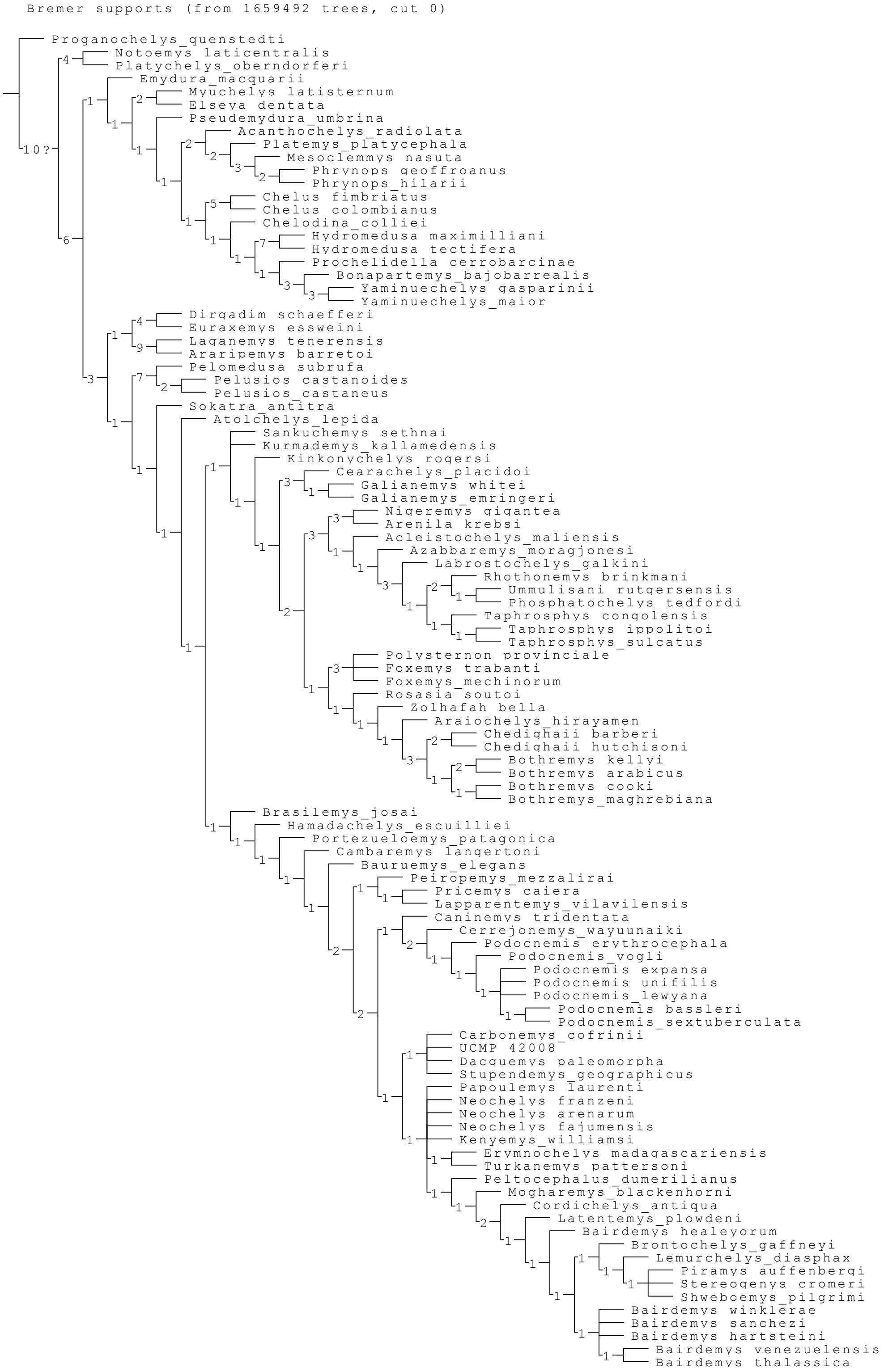

Figure S2-6. Bremer support values using TBR from existing trees retaining suboptimal trees by 10 steps

4. List of common synapomorphies for Stereogenyina

Mogharemys blackenhorni:
All trees:
Char. 53: 0 --> 1
Char. 113: 0 --> 1
Char. 117: 1 --> 0

Brontochelys gaffneyi:
All trees:
No autapomorphies:

Lemurchelys diasphax:
All trees:
Char. 110: 1 --> 0

Shweboemys pilgrimi:
All trees:
No autapomorphies:

Stereogenys cromeri:
All trees:
Char. 129: 1 --> 2
Some trees:
Char. 42: 1 --> 0

Cordichelys antiqua:
All trees:
Char. 8: 0 --> 1
Char. 32: 1 --> 0
Char. 76: 0 --> 1
Char. 169: 0 --> 1
Char. 181: 2 --> 1
Char. 182: 1 --> 2
Char. 192: 1 --> 0
Char. 205: 0 --> 1
Some trees:
Char. 233: 1 --> 0

Latentemys plowdeni:
All trees:
Char. 40: 1 --> 0
Char. 245: 1 --> 2

Bairdemys thalassica:
All trees:
Char. 102: 0 --> 1

Bairdemys healeyorum:
All trees:
Char. 180: 0 --> 1
Char. 241: 1 --> 0

Bairdemys venezuelensis:
All trees:
No autapomorphies:

Bairdemys hartsteini:
Some trees:
Char. 124: 1 --> 0

Bairdemys sanchezi:
All trees:
Char. 17: 1 --> 2

Char. 110: 1 --> 0
Some trees:
Char. 11: 1 --> 0
Char. 18: 0 --> 1
Char. 40: 1 --> 0
Char. 58: 1 --> 0
Char. 129: 1 --> 2

Bairdemys winklerae:
Some trees:
Char. 18: 0 --> 1

Piramys auffenbergi:
All trees:
Char. 8: 0 --> 1
Some trees:
Char. 119: 0 --> 1

Node 179:
All trees:
Char. 73: 0 --> 1
Char. 78: 0 --> 1
Char. 110: 0 --> 1

Node 180:
All trees:
Char. 53: 0 --> 1

Node 181:
All trees:
Char. 140: 1 --> 0

Node 182:
All trees:
Char. 88: 0 --> 1

Node 183:
All trees:
Char. 90: 0 --> 1

Node 184:
All trees:
Char. 54: 1 --> 0
Char. 55: 1 --> 2
Char. 69: 0 --> 1
Char. 74: 1 --> 2
Char. 75: 0 --> 1
Char. 89: 0 --> 1
Char. 127: 0 --> 1

Node 185:
All trees:
Char. 76: 0 --> 1

Node 186:
Some trees:
Char. 32: 1 --> 0
Char. 77: 0 --> 2
Char. 79: 0 --> 1
Char. 114: 1 --> 2
Char. 245: 1 --> 2

Node 187:
All trees:
Char. 77: 0 --> 1
Char. 87: 1 --> 0

Some trees:
Char. 129: 1 --> 2

Node 188:
All trees:
Char. 34: 0 --> 1
Char. 35: 0 --> 1
Some trees:
Char. 11: 0 --> 1
Char. 58: 0 --> 1
Char. 70: 12 --> 0
Char. 124: 0 --> 1
